# Hemostatic Alginate/Nano-Hydroxyapatite Composite Aerogel Loaded with Tranexamic Acid for the Potential Protection against Alveolar Osteitis

**DOI:** 10.3390/pharmaceutics14102255

**Published:** 2022-10-21

**Authors:** Mai El Halawany, Randa Latif, Mohamed H. H. AbouGhaly

**Affiliations:** 1 Department of Pharmaceutics and Industrial Pharmacy, Faculty of Pharmacy, Cairo University, Kasr El-Ainy Street, Cairo 11562, Egypt; 2 Department of Pharmaceutics and Industrial Pharmacy, School of Pharmacy, Newgiza University, Km. 22 Cairo-Alex Road, Giza P.O. Box 12577, Egypt

**Keywords:** aerogel, hemostasis, tranexamic acid, nano-hydroxyapatite, alveolar osteitis, alginate

## Abstract

Wound control in patients on anticoagulants is challenging and often leads to poor hemostasis. They have a higher tendency to develop alveolar osteitis after tooth extraction. The application of a hemostatic dressing that has a high absorbing capacity and is loaded with an antifibrinolytic drug could help in controlling the bleeding. Alginate/nano-hydroxyapatite (SA/Nano-HA) composite aerogels loaded with tranexamic acid (TXA) were prepared. Nano-HA served as a reinforcing material for the alginate matrix and a source of calcium ions that helps in blood clotting. It influenced the porosity and the water uptake capacity. TXA release from SA/Nano-HA aerogels showed a biphasic profile for up to 4 h. Blood coagulation studies were performed on human whole blood. The TXA-loaded aerogel significantly reduced the clotting time by 69% compared to the control (*p* < 0.0001). Recalcification time was significantly reduced by 80% (*p* < 0.0001). Scanning electron microscopy analysis revealed the porous nature of the aerogels and the ability of the optimum aerogel to activate and adhere platelets to its porous surface. The cell migration assay showed that there was a delay in wound healing caused by the TXA aerogel compared to the control sample after treating human fibroblasts. Results suggest that the developed aerogel is a promising dressing that will help in hemostasis after tooth extraction.

## 1. Introduction

Alveolar osteitis (dry socket) is a common complication of tooth extraction. It occurs as a result of the degradation of the blood clot at the site of the tooth extraction before complete wound healing [1]. It is usually accompanied by inflammation of the alveolar bone, resulting in strong pain and delayed wound healing [2]. During tooth extraction, the formation of a blood clot is important. It acts as a shield or physical barrier over the fundamental bone and nerve endings in the vacant tooth hole and prevents movement of bacteria into nearby healthy tissues. It also provides the foundation for the growth of new tissues [3]. The incidence of alveolar osteitis is 5.6% after tooth extraction, and the incidence rises to up to 30% after surgical extraction of third molars [2].

The management of alveolar osteitis includes the treatment of symptoms. Systemic or topical analgesics and mouthwashes were suggested for the relief of pain and swelling. The guidelines discourage the prescription of antibiotics unless evidence of bacterial infection to avoid bacterial strain resistance and hypersensitivity [1].

Prevention of alveolar osteitis is the key method. However, the prevention technique remains a debated topic as no single technique has gained worldwide acceptance [4]. Prevention methods include the use of a topical 0.12–0.2% chlorohexidine rinse or gel alone or combined with antibiotics [5,6], and the topical application of tranexamic acid, and other antifibrinolytic agents peri- and postoperative tooth extraction to stabilize the formed blood clot and prevent its dissolution [7]. Utilization of irrigations and mouthwashes are temporarily effective due to rapid wash out from the site of application.

Patients taking anticoagulant therapy suffer from postdental extraction complications. Administration of anticoagulants leads to the collapse or dissolution of the blood clot; hence, alveolar osteitis may develop. Moreover, anticoagulants increase the hazards of uncontrolled bleeding [8]. Stopping the anticoagulant therapy before tooth extraction could control bleeding, but it might increase the risk of thromboembolism. Therefore, the use of local hemostatic agents after tooth extraction would enhance the safety of patients treated with an anticoagulant [9,10].

Tranexamic acid (TXA), a trans-4-aminomethyl cyclohexane carboxylic acid, is a lysine analogue. It acts as a competitive inhibitor that prevents the conversion of plasminogen into plasmin by reversibly binding four to five lysine receptors on plasminogen. It is used as an antifibrinolytic agent, helps in the construction of the fibrin mesh-network, and withstands its corruption [11,12].

Tranexamic acid is administered orally and through an intravenous (IV) route to control menorrhagia and systemic bleeding. It has a bitter taste and gastrointestinal side effects if used in a dose of more than 3 g/day. The IV route is used in surgeries, but it has a thromboembolism risk [13].

In dental procedures, TXA is used as a socket irrigation solution to avoid postextraction bleeding. This method of application has limited accessibility, poor efficiency, and poor control over the delivered dose due to the rapid wash out of the drug from the application area [14].

For this purpose, the topical application of TXA in the form of a solid dressing provides local delivery of the drug and acts as a dental socket plug that restricts bleeding and accelerates wound healing.

Aerogels are microporous solids in which the dispersed phase is a gas [15].They are produced by drying the fluid part of the wet gel and replacing it with a gas using a suitable drying technique. The resulting aerogel matrix is porous and ultralight, and made of crosslinked 3D networks, partially like the physical characteristics of the natural extracellular matrix. This structure provides mechanical support for cell adhesion, proliferation, and other cellular functions [16,17]. The porous structure is also advantageous for the absorption of biological fluids; hence, reducing wound exudates and enhancing blood clot formation [15]. Thus, aerogels can be considered as suitable dressings for postdental operations, either in the plain or medicated form.

Macromolecules (such as chitosan, alginate, and gelatin), inorganic and composite materials were used to develop aerogels [18,19,20].

Alginates have been extensively studied as hemostatic materials due to their high absorbency and hydrophilicity. They have a positive impact on wound healing and epidermal regeneration [21]. Alginates are natural polymers extracted from marine brown algae. They are biocompatible, biodegradable, and nontoxic under physiological conditions. Alginate crosslinking with divalent cations, such as calcium, leads to stiff gel formation. Additionally, calcium alginate gel activates platelet and blood coagulation; hence, it could be used as a hemostatic dressing [22].

The incorporation of nano-hydroxyapatite (Nano-HA) with alginate has been investigated [23,24]. Nanocomposite films comprising Nano-HA and alginate with antibacterial activity were applied in tissue engineering [25], and nanocomposite scaffolds of Nano-HA and alginate were also prepared for bone tissue engineering [26,27]. Nano-HA is an inorganic material with a similar chemical composition and structure as the mineral phase of human bones and teeth [28]. It is biocompatible and osteoconductive, helps in cell adhesion and proliferation, and regulates bone defects. It releases calcium ions that help in hemostasis [29].

In this work, we present an alginate/nano-hydroxyapatite (SA/Nano-HA)-based composite aerogel loaded with TXA. Through the hemostatic effect of TXA, along with the polymeric matrix, the formulation is expected to help with bleeding cessation after tooth extraction. Moreover, a decrease in the risk of alveolar osteitis is expected. Nano-HA has an additional role in biomineralization and decreasing the risks of bone defects. A full factorial design will be applied to study the effect of different formulation variables on the developed aerogel. The behavior of the prepared formulations in simulated biological fluids (SBFs), their swelling rate, and drug release will be examined. The optimum aerogel will be examined using scanning electron microscopy, Fourier transform infrared spectroscopy (FTIR), and blood coagulation studies to determine its hemostatic activity using human whole blood. An indirect cell migration assay using normal human fibroblast cell lines will be performed to check for its effect on the wound healing process.

## 2. Materials and Methods

### 2.1. Materials

Tranexamic acid (TXA) was a gift from Amoun Pharmaceuticals Company, Al Qalyubia, Egypt. Nano-hydroxyapatite (Nano-HA) (nano-powder, <200 nm, BET > 97%) and alginic acid sodium salt (SA) (high viscosity) were purchased from Sigma-Aldrich, St. Louis, MO, USA. Tris base and ninhydrin were purchased from the Fisher Scientific company, Loughborough, UK. Glutaraldehyde (25% aqueous solution) was purchased from Oxford Laboratory Fine Chemicals LLP, Maharashtra, India. The normal human fibroblast cell line was obtained from Nawah Scientific, Cairo, Egypt. Cutanplast^®^, a porous reabsorbable gelatin sponge (1 × 1 × 1.5 cm) with a hemostatic effect, was purchased from Mascia Brunelli Spa, Milan, Italy. Calcium chloride, magnesium chloride, sodium chloride, potassium chloride, dipotassium hydrogen phosphate, and sodium sulphate were of analytical grade.

### 2.2. Methodology

#### 2.2.1. Preparation of Simulated Body Fluid

Li et al. [30] reported that there was a difference in the release profile of TXA when they ran the experiment in phosphate-buffered saline (PBS, pH 7.4) and anti-coagulated blood plasma. They attributed this variability to the difference in ionic strength. Therefore, SBF, with a similar pH and ionic strength to that of human blood, was used in the fluid uptake and release studies in our work.

The SBF was made of 8.04 g of NaCl, 0.36 g of NaHCO_3_, 0.23 g of KCl, 0.23 g of K_2_HPO_4_·3H_2_O, 0.31 g of MgCl_2_·6H_2_O 39 mL of 1.0 M HCl, 0.29 g of CaCl_2_, 0.07 g of Na_2_SO_4_, and 6.12 g of Tris, which was added to 900 mL deionized water. Hydrochloric acid (1 M) was used to adjust the pH value to 7.4, and the volume was adjusted to 1 L.

#### 2.2.2. Preparation of Alginate/Nano-Hydroxyapatite Composite Aerogel Loaded with Tranexamic Acid

##### Experimental Design

A two-level, three-factor full factorial design (2^3^ FFD) was employed. The independent variables and the levels of each variable are shown in Table 1.

##### Method of Preparation

Aerogels were prepared by dispersing specified amounts of SA in deionized water. Nano-HA was dispersed in deionized water and stirred at 1200 rpm for 30 min at ambient temperature. Nano-HA dispersion was added slowly to the alginate, stirred for 2 h until complete homogeneity was reached, and then was sonicated for 5 min [25]. The resulting mixture was poured into circular molds with a 1.8 cm diameter and 0.6 mm depth. Some formulations were crosslinked using 1 mL of 0.5 M CaCl_2_ (Table 2). TXA (20 mg) was then added while stirring until complete homogeneity was reached [31]. All aerogel formulations were frozen at −21 °C overnight, then freeze-dried at −60 °C, 0.1 atm for 24 h [32].

#### 2.2.3. In Vitro Characterization of Alginate/Nano-Hydroxyapatite Composite Aerogel Loaded with Tranexamic Acid

##### Drug Content

Samples (equivalent to 20 mg TXA, *n* = 3) were weighed and placed in 50 mL of deionized water under magnetic stirring for 24 h. Aliquots were filtered through a 0.22 μm Millipore^®^ filter, diluted, and assayed at a λ max of 565 nm using a UV Spectrophotometer (Shimadzu UV 1601 PC, Kyoto, Japan). Percent drug content (% DC) was calculated using Equation (1).

(1) $$\%\;\mathrm{DC}=\frac{\mathrm{Actual}\;\mathrm{TXA}\;\mathrm{content}}{\mathrm{Theoretical}\;\mathrm{TXA}\;\mathrm{content}}\times 100$$

##### Matrix Porosity

Porosity of the aerogels was determined using a helium porosimeter. Grain and bulk densities were calculated by dividing the aerogel weight (Sartorius TE 124S, Sartorius Lab Instruments GmbH & Co. KG, Göttingen, Germany) by the grain and bulk volumes, respectively. Bulk volume was calculated from the dimensions of the aerogels measured by a digital caliper. A helium pycnometer (Ultrapyc 1200e Quantachrome pycnometer, Anton Paar GmbH, Graz, Austria) was used to determine the grain volumes of the aerogels [33]. Porosity was calculated using Equation (2).

(2) $$\emptyset =\frac{\rho_{g\;}-{\;\rho }_{b}}{\rho_{g}}\times 100$$

where ∅ is the porosity, and $\rho_{g\;}$ and $\rho_{b}$ are the grain and the bulk densities, respectively. The test was run in triplicate.

##### Swelling Properties

The fluid uptake or swelling capacity of the prepared aerogels was determined in the SBF. Briefly, the aerogels were weighed and immersed in 10 mL of SBF (pH 7.4) at 37 °C. The swelled aerogels were taken out of the buffer after soaking for 5, 15, 30, 60, 120, and 240 min. They were placed on a glass plate inclined at 45° from the surface for 1 min to remove excess SBF. The swelled aerogels were then reweighed. The fluid uptake % in the SBF was calculated using Equation (3).

(3) $$\mathrm{Swelling}\;\%=\frac{\left(W_{t}-W_{0}\right)}{W_{0}}\times 100$$

where W_t_. is the weight of the aerogel at a given time, t, during swelling and W_0_ is its initial weight. The fluid uptake profile over 4 h and the maximum amount of fluid uptake were determined. The test was run in triplicate.

Additionally, the swelling properties of the optimum aerogel in blood were obtained and the results were compared to that of the SBF.

##### In Vitro Release of Tranexamic Acid

The in vitro release profile of TXA from SA/Nano-HA composite aerogels was evaluated in the SBF (pH 7.4). The aerogels were incubated in 10 mL of SBF and placed in a thermostatic shaking water bath at 37 °C under constant stirring at 80 rpm [34,35].

Samples were withdrawn at 5, 15, 30, 60, 120, 180 and 240 min, and replaced with the same volume of fresh medium (0.5 mL). The samples were analyzed for TXA content by spectrophotometric analysis at a λ max of 565 nm [36]. The percentage of drug released was calculated and plotted versus time. The test was run in triplicate.

##### Linear Regression Analysis

The release data were subjected to linear regression analysis to determine the order of release. Data were fitted to zero-order, first-order and Higuchi diffusion models [37]. The model with the highest coefficient of determination (r^2^) was considered the order of best fit.

##### Fourier Transform Infrared Spectroscopy

Fourier transform infrared spectroscopy (FTIR) was performed to check for possible interactions between the formulations’ components. A fixed amount (2.5 mg) of sodium alginate, nano-hydroxyapatite, tranexamic acid, and the optimum formulation was mixed with dry potassium bromide. Each mixture was compressed into a small disc and scanned using the FTIR spectrophotometer (Shimadzu IR-345-U-04, Kyoto, Japan) over 4000–400 cm^−1^ with an S/N ratio of 30,000:1 for a total of 32 scans.

##### Scanning Electron Microscopy

The surface topography, together with the internal morphology of the nonmedicated calcium alginate aerogel and nonmedicated SA/Nano-HA aerogel, was examined using scanning electron microscopy (SEM). The optimized system was also scanned before and after swelling and drying at room temperature. Samples were treated using a TESCAN scanning electron microscope (VEGA3 SBU, Brno, Czech Republic) after coating with a thin layer of gold for 180 s using the Quorum Techniques Ltd. sputter coater (Q150t, Lewes, UK). Imaging was performed at different magnification powers.

#### 2.2.4. Blood Coagulation Study

##### Assessment of Whole Blood Clotting Time

Clotting time (CT) was determined for the optimum aerogel containing 20 mg of TXA, the plain aerogel without TXA, the TXA solution containing 20 mg of TXA, and Cutanplast^®^ as the control. Human blood was collected from healthy volunteers and anticoagulated with acid citrate dextrose (20 mM citric acid, 110 mM sodium citrate, and 5 mM D-glucose). Test tubes containing 1 mL of whole blood were heated at 37 °C for 10 min. The aerogel/control solution was added to the test tubes, followed by 0.05 mL of 0.2 M CaCl_2_. The tubes were inverted every 10 s and held in position for 1 s before reverting. CT was the time when no flow of blood was observed after the tube was inverted. The test was run in triplicates [38].

##### Plasma Recalcification Time

Plasma recalcification time (RT) was determined for the optimum aerogel with and without TXA, along with the TXA solution and Cutanplast^®^, following Hu et al.’s method [39] with modifications. Briefly, anticoagulated blood was centrifuged at 5200 rpm for 15 min to obtain poor platelet plasma (PPP). Next, 0.5 mL of PPP was incubated at 37 °C with the aerogel/control solution for 10 min, and then, 30 μL of 0.2 M CaCl_2_ was added. Plasma was observed every 30 s for any appearance of turbidity (white jelly), which reflects fibrin thread formation. The test was run in triplicates.

##### Platelet Adhesion

Platelet adhesion was performed as described by Fatahian et al. [31]. Platelet-rich plasma (PRP) was obtained by centrifugation of the whole blood treated with an acid citrate dextrose mixture as the anticoagulant at 2500 rpm for 5 min. The optimum aerogel was washed thrice with deionized water, followed by a rinse with 0.1 M PBS. PRP (0.5 mL) was dropped onto the aerogel surface and left for 20 min at 37 °C. The aerogel was further washed to eliminate plasma proteins and nonadhered platelets with 0.1 M PBS, and then reacted with 2% glutaraldehyde solution for 1 h to fix adhered platelets. It was washed again with PBS, dehydrated using ethanol, and left to dry overnight. Platelet adherence to the sample was analyzed using SEM.

##### In Vitro Dynamic Blood Clotting Index

The dynamic blood clotting index (BCI) was performed on the optimum aerogel with and without TXA, with the TXA solution, and with Cutanplast^®^. An anticoagulated blood sample was used as a negative control, according to Catanzano et al.’s method [34]. Briefly, whole blood was collected and anticoagulated by mixing with the acid citrate dextrose mixture. The anticoagulated blood (1 mL) was dropped on the surface of the aerogels in a 50 mL glass beaker. Once the aerogels were completely covered, blood coagulation was initiated by adding 50 µL of the 0.2 M CaCl_2_ solution, followed by 10 min of incubation at 37 °C under gentle shaking. Red blood cells (RBCs) that were not stuck in the clot were hemolyzed with 25 mL of deionized water. Water was added by dripping it down the inside wall of the beakers without disturbing the blood clot. The absorbance (A) of blood sample solutions was measured at 542 nm using a spectrophotometer. The % BCI of anticoagulated whole blood in 25 mL of deionized water was assumed to be 100% as a reference value. The % BCI of the blank and the samples were computed according to Equation (4).

(4) $$\%\;\mathrm{BCI}\;=\frac{A_{1}\;\mathrm{of}\;\mathrm{hemolyzed}\;\mathrm{blood}}{A\;\mathrm{of}\;\mathrm{anticoagulated}\;\mathrm{whole}\;\mathrm{blood}}\times 100$$

where A_1_ and A are the absorbance of unbounded blood hemolyzed by water after contacting samples and anticoagulated whole blood at λ 542 nm, respectively. The test was run in triplicate.

#### 2.2.5. Indirect Cell Migration Assay

The wound healing assay using the wound scratch technique was carried out on the normal human fibroblast cell line (HSF). Cells were maintained in Dulbecco’s Modified Eagle Medium (DMEM) and supplemented with 100 mg/mL of streptomycin, 100 units/mL of penicillin, and 10% heat-inactivated fetal bovine serum (FBS) in a humidified 5% (*v*/*v*) CO_2_ atmosphere at 37 °C.

Cells were plated at a density of 2 × 10^5^ cells per well onto a coated 12-well plate for the scratch wound assay and cultured overnight in 5% FBS-DMEM at 37 °C and 5% CO_2_. On the next day, horizontal scratches were introduced into the confluent monolayer. The plate was washed thoroughly with PBS and the control wells were replenished with fresh medium, whereas the formulation wells were treated with fresh media containing aerogel extract [40]. The aerogel extract was prepared as reported in the literature [41]. Briefly, 0.1 gm of the aerogel was extracted in 10 mL of DMEM for 24 h at 37 °C in an environment of 5% CO_2_ (adapted from ISO 10993-12). Then, the 100 µL extract, completed to 1 mL with the culture medium, was added to each well-plate. Images were taken using an inverted microscope at the predetermined time points. The plate was incubated at 37 °C and 5% CO_2_ in-between time points. The acquired images were analyzed via MII ImageView software (version 3.7, PT Mitra Integrasi Informatika, Jakarta, Indonesia) to detect wound width. Wound width was plotted as a function of time and the cell migration rate was calculated from slopes of the produced plots [36]. The test was run in triplicate.

#### 2.2.6. Statistical Analysis

A factorial ANOVA was used to analyze the 2^3^ full factorial design and desirability was calculated to choose the optimum formulation using the Design-Expert^®^ software (Version 9, Stat-Ease Inc., Minneapolis, MN, USA).

An unpaired *t*-test and one-way analysis of variance (one-way ANOVA) were applied to analyze blood coagulation tests and indirect cell migration assay data using the GraphPad Prism software (version 9.3.1, Boston, MA, USA). Differences were significant at *p*-values < 0.05.

## 3. Results and Discussion

### 3.1. Physical Properties of the Prepared Alginate/Nano-Hydroxyapatite Aerogels Loaded with Tranexamic Acid

SA and Nano-HA were used to prepare eight aerogels loaded with TXA (Table 2). All aerogels were cylindrical in shape and white in color with an average diameter of 1.8 ± 0.05 cm and thickness of 0.6 ± 0.04 cm. They had a smooth surface with a porous structure that remarkably decreases upon increasing the amount of Nano-HA (Figure S1).

### 3.2. Drug Content

The calculated % DC in the formed aerogels ranged from 91 ± 0.4% to 94 ± 0.7%, indicating the suitability of the formulation parameters which avoided drug loss during the aerogel preparation.

### 3.3. Matrix Porosity

Porosity refers to the volume that the void spaces occupy out of the total volume of the material. High porosity reflects a high surface area/volume ratio. It favors the fast release of drugs and promotes hemostasis by facilitating cell adhesion and entangling RBCs, plasma, and other anticoagulant factors [42]. Porosity of the developed aerogels ranged from 91.96 ± 0.16 to 96 ± 0.04%. This indicated that the fabrication process with SA and Nano-HA favored the formation of aerogels with high porosity.

The statistical analysis revealed that increasing the concentration of alginate significantly increased porosity, whereas Nano-HA significantly decreased the porosity (*p* < 0.0001) (Figure S2A,B). The addition of Nano-HA made the pores more interconnected with denser and thicker pore walls [27]. Crosslinking SA with Ca^+2^ ions also significantly decreased the porosity of aerogels (*p* < 0.0001) (Figure S2C). Alginate crosslinking gives a compact matrix that resists the free motion of molecules. Therefore, during the freezing phase, smaller ice crystals are formed as the polymer matrix opposes their growth, leaving smaller pore volumes and thicker walls after sublimation [42].

### 3.4. Determination of Swelling Properties

The capability of a hemostatic dressing to absorb high volumes of fluids is an important aspect of their functionality. The fast uptake of blood fluids helps the hemostasis process. This is achieved by concentrating the anticoagulant factors and tangible elements, such as platelets and RBCs, which enhances the rapid clotting of blood [43,44]. Fluid uptake depends on the hydrophilicity of the used polymer and porosity of the dressing matrix [45].

**Figure 1:**
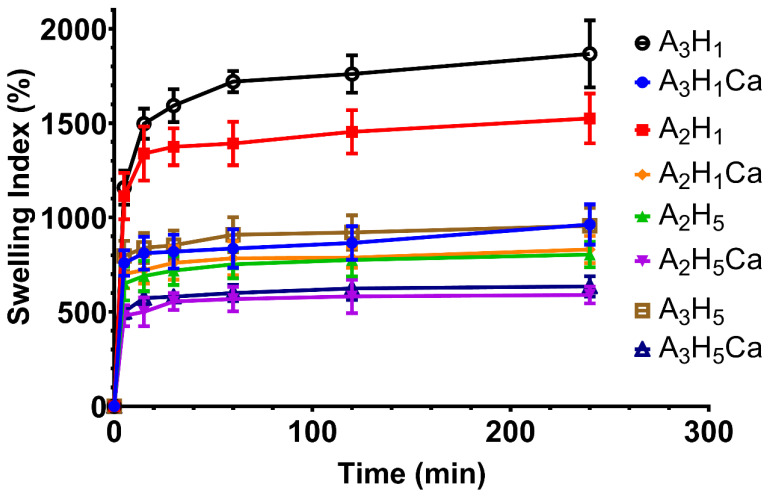
Swelling rates of alginate/nano-hydroxyapatite aerogels loaded with tranexamic acid in simulated body fluid (pH 7.4).

The swelling profiles of the prepared aerogels were determined (Figure 1). The different aerogels showed a similar trend where a rapid fluid uptake was observed within the first 5–15 min, followed by equilibrium. A_3_H_1_ and A_2_H_1_ aerogels reached equilibrium in approx. 15 min and had the highest extent of swelling. Both aerogels had the lowest amount of Nano-HA and Ca^+2^ ions in their backbone. Meanwhile, the higher concentration of SA in the A_3_H_1_ aerogel relative to A_2_H_1_ contributed to its higher extent of swelling. This might be due to the hydrophilic nature of alginate. The presence of hydroxyl and carboxyl groups in mannuronic acid (M) and guluronic acid (G) monomers of the alginate skeleton bind to water molecules via hydrogen bonding. The saturation of the bonding sites with water leads to equilibrium [43]. A_3_H_1_ showed the highest extent of fluid uptake (1867 ± 90%), whereas A_2_H_5_Ca possessed the least (590 ± 45%).

Statistical analysis showed that increasing the concentration of SA in aerogels significantly increased swelling capacity (*p* = 0.0003) (Figure S3A). This is due to the hydrophilicity of SA and the higher number of water bonding sites (hydroxyl and carboxyl groups) in the matrix with increasing SA concentration [32]. Increasing the amount of Nano-HA significantly decreased swelling (*p* < 0.0001) (Figure S3B). Nano-HA decreased the porosity of the prepared aerogels as the pore walls became denser and thicker. They might also have blocked attachment sites on SA by steric hindrance and formed a physical barrier between water and SA [46].

Ionic crosslinking of SA using Ca^+2^ ions significantly decreased swelling (*p* < 0.0001) (Figure S3C). The divalent ions link with ionic bonds to the free carboxyl groups in the G residues of SA to form an egg-box conformation hydrogel matrix. This matrix is more compact and rigid and less porous, leading to lower fluid uptake [47].The crosslinking does not allow the free movement of the polymer chains away from each other to incorporate water into the matrix [48].

### 3.5. In Vitro Drug Release of TXA from Alginate/Nano-Hydroxyapatite Aerogels

**Figure 2:**
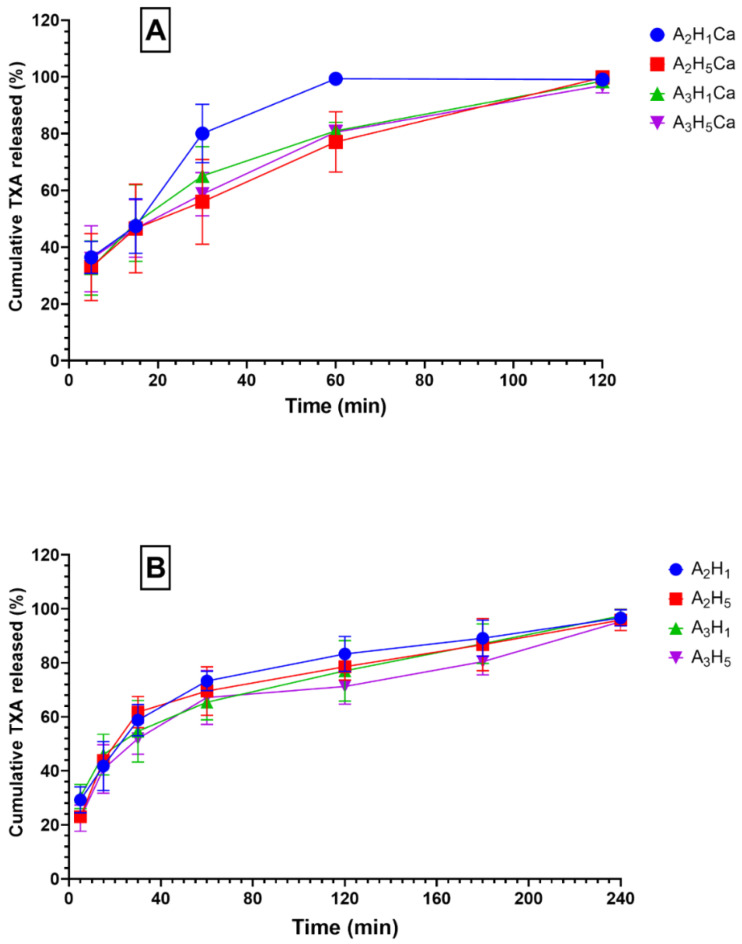
Cumulative release profile of tranexamic acid (TXA) in simulated body fluid; (**A**) monophasic release pattern of A_2_H_1_Ca, A_2_H_5_Ca, A_3_H_1_Ca, and A_3_H_5_Ca aerogels; (**B**) biphasic release pattern of A_2_H_1_, A_2_H_5_, A_3_H_1_, and A_3_H_5_ aerogels.

Figure 2 shows the cumulative drug release profile of TXA from the different aerogels. The in vitro release showed that all the aerogels had an initial burst release. The initial fast release was due to their high porosity and fast swelling kinetics, which made TXA peak within 30 min [34]. Additionally, TXA water solubility contributed to its fast dissolution and diffusion from the aerogels once it came in contact with the release medium. The differences in pore size did not affect the release due to the high porosity of both matrices. In the next phase, the porous matrix swelled, and a gel layer was formed that could control the release rate. However, due to differences in polymer crosslinking, the formed gels were of different properties.

A biphasic release of TXA is seen for A_2_H_1_, A_2_H_5_, A_3_H_1_, and A_3_H_5_ over 4 h. More than 50% of TXA was released within 30 min, followed by a sustained-release phase. The initial phase followed the Higuchi diffusion mechanism, whereas the sustained-release phase followed zero-order kinetics. Table S1 shows the kinetic parameters of the prepared aerogel release profile. These formulations were prepared without Ca^+2^ crosslinking. They had a high swelling capacity and were followed by the formation of a gel structure that was able to control TXA release. The sustained-release regimen was probably due to the in situ crosslinking of the free carboxyl groups in G residues of the alginate skeleton with Ca^+2^ ions present in the release medium. TXA molecules became trapped inside the egg-box structure and needed further time to be released from the matrix [49]. That is why the further release proceeded at a much lower rate, giving rise to a biphasic profile.

The biphasic release phenomenon is beneficial, because the hemorrhage needs instant high doses of TXA to initiate inhibition of the plasminogen-converting mechanism and to favor fibrin formation to start blood clotting. The sustained release of TXA over 4 h is advantageous in stabilizing the formed clot [38,45].

A monophasic drug release pattern was seen for A_2_H_1_Ca, A_2_H_5_Ca, A_3_H_1_Ca, and A_3_H_5_Ca. The linear regression analysis revealed that the TXA release followed the Higuchi diffusion mechanism. Aerogels crosslinked with Ca^+2^ ions had a low swelling capacity compared to the non-crosslinked aerogels and continued to release TXA at the same rate until it released more than 95% of TXA in 2 h. This enabled them to release the water-soluble drug at a relatively high rate when exposed to the medium.

Statistical analysis showed that neither changing the concentration of alginate nor nano-hydroxyapatite had a significant effect on the release pattern (Figure S4A,B).

### 3.6. Selection of the Optimized Formula

The optimum formula was chosen based on the desirability values calculated by Design-Expert^®^. The A_3_H_1_ aerogel (its composition is listed in Table 2) had the highest desirability (0.897), and it possessed the highest swelling %, highest porosity %, and a biphasic release profile. Therefore, it was selected for further investigation.

### 3.7. Fourier Transform Infrared Spectroscopy

FTIR spectra of A_3_H_1_ and its individual components are shown in Supplementary Figure S5. Characteristic peaks of each component appeared in the optimum aerogel and confirmed partial crosslinking of alginate with Ca^+2^ ions released from Nano-HA by conserving TXA molecules without any molecular interactions. More data are supplied in the Supplementary Materials.

### 3.8. Scanning Electron Microscopy

**Figure 3:**
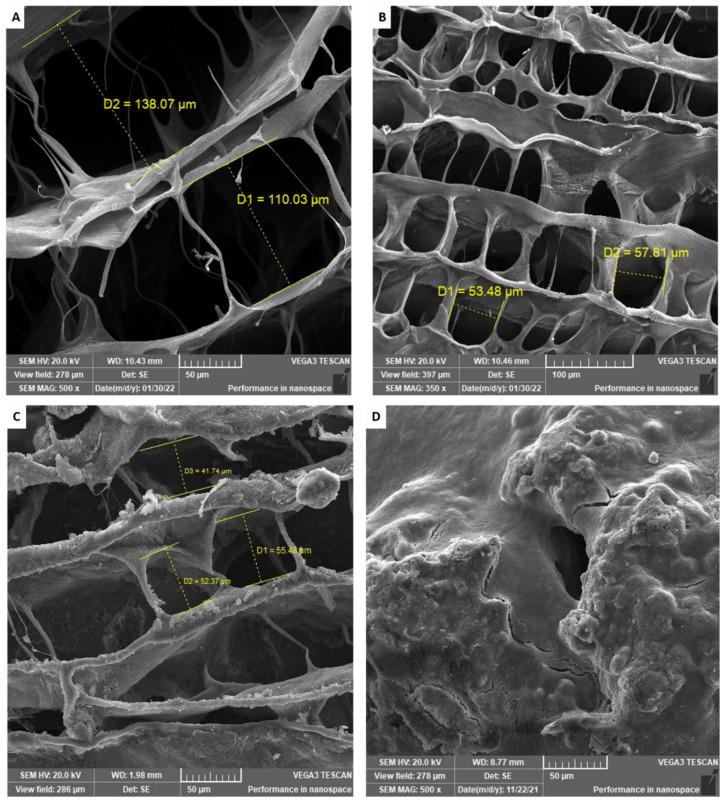
Scanning electron microscope images of (**A**) plain alginate aerogel; (**B**) plain alginate/nano-hydroxyapatite aerogel (A_3_H_1_N); (**C**) alginate/nano-hydroxyapatite aerogel loaded with tranexamic acid (A_3_H_1_); (**D**) A_3_H_1_ hydrogel after drying.

Scanning electron microscopy was used to explore the internal and external morphology of the optimum aerogel. The nonmedicated calcium alginate aerogel (Figure 3A) had very wide pores, with a diameter ranging from 110 to 140 µm. The pore walls were very thin with a smooth surface.

In contrast, the nonmedicated SA/Nano-HA aerogel, A_3_H_1_N, (Figure 3B) and the TXA-loaded SA/Nano-HA aerogel, A_3_H_1_, (Figure 3C) showed narrower pores, with a diameter ranging from 50 to 80 µm. The pore walls appeared to be thicker and denser due to the deposition of hydroxyapatite nanoparticles that supported and gave the reinforcing effect to the aerogel. The roughness of the surface of the matrix (Figure 3C) might be due to the deposition of TXA particles on its walls.

The morphology of the A_3_H_1_ hydrogel after swelling and drying (Figure 3D) showed solid rough surfaces with many corrugations. The porous structure disappeared due to swelling of the alginate matrix and entanglement of the chains, followed by the collapse of the internal structure after air drying.

### 3.9. Blood Coagulation Studies

Hemostasis is a complex procedure that involves platelets, specific blood cells, and a variety of molecules, resulting in the development of insoluble fibrin threads [50]. Thus, there are numerous indicators for the in vitro examination of hemostatic effects, including the clotting time (CT), plasma recalcification time (RT), dynamic blood clotting index (BCI), platelet adhesion, and aggregation rate.

#### 3.9.1. Assessment of Whole Blood Clotting Time

**Figure 4:**
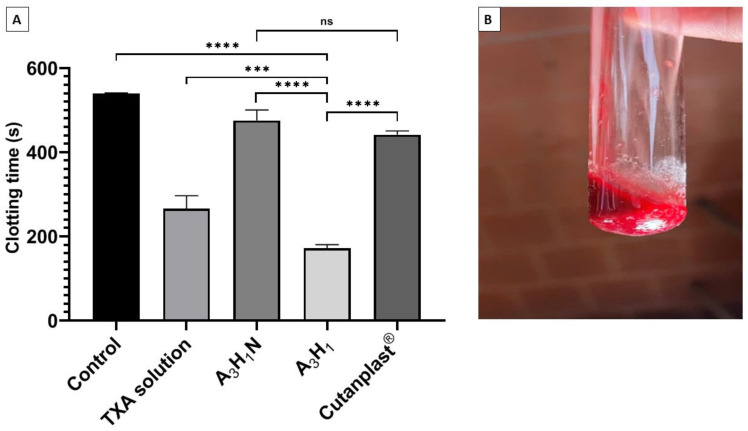
(**A**) Blood clotting time test for the optimum aerogel with tranexamic acid (A_3_H_1_) and without tranexamic acid (A_3_H_1_N), tranexamic acid solution, Cutanplast^®^, and negative control (without any material added) and (**B**) clotting test for A_3_H_1_. ns = *p* > 0.05, *** = *p* ≤ 0.001, and **** = *p* ≤ 0.0001.

The CT determination gives an indication about the entire process of clotting, including for blood cells and plasma. Statistical analysis showed that there was a significant difference between the CT of each of the tested samples and the control group. The CT of the unmedicated aerogel (A_3_H_1_N) significantly decreased to 475 ± 25 s from 540 ± 23 s for the control sample (*p* ≤ 0.01), whereas it was non-significantly different from Cutanplast^®^, which had a CT of 442 ± 9 s (*p* = 0.2467) (Figure 4A). This clearly demonstrated the hemostatic effect of the SA/Nano-HA aerogel due to its hydrophilicity and porosity. When the aerogel is in contact with blood, it absorbs blood and entangles RBCs and other blood cells in the porous matrix and on the surface. Therefore, it concentrates the coagulation factors. This helps in forming hydrogel-blood aggregates (Figure 4B) and in the formation of blood clots, even in the absence of TXA [38,50].

The tranexamic-acid-loaded aerogel significantly decreased the CT compared to the unmedicated aerogel (*p* ≤ 0.0001) (Figure 4A). TXA has a plasminogen-converting inhibition mechanism. Thus, it blocked the conversion of plasminogen into plasmin and hastened coagulation. Additionally, its antifibrinolytic effect supported the fibrin threads’ development and stabilized the formed clots [11]. The TXA-loaded aerogel showed a significantly lower CT compared to the TXA solution (*p* ≤ 0.001) (Figure 4A). This might be attributed to the additive effect of both TXA and the hemostatic porous aerogel.

#### 3.9.2. Plasma Recalcification Time

**Figure 5:**
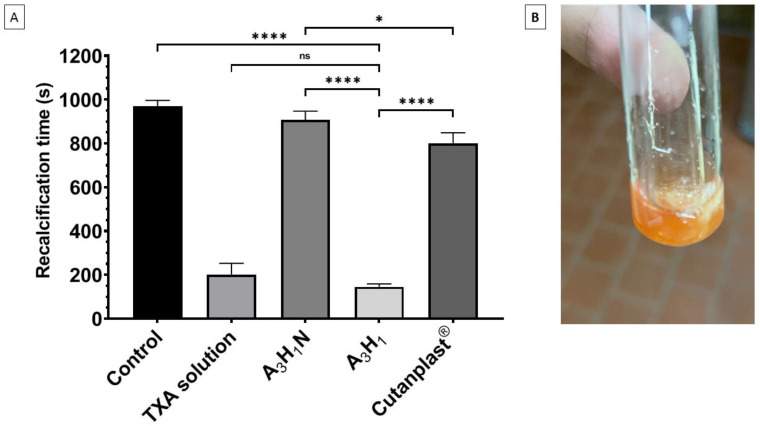
(**A**) Recalcification time of optimum aerogel with tranexamic acid (A_3_H_1_) and without tranexamic acid (A_3_H_1_N), and with tranexamic acid solution, Cutanplast^®^, and a negative control (without any material added); (**B**) recalcification test of A_3_H_1_ aerogel. ns = *p* > 0.05, * = *p* ≤ 0.05, and **** = *p* ≤ 0.0001.

The plasma recalcification time test gives an indication about the stimulation of the intrinsic coagulation pathway that supports fibrin thread formation [51]. It is used for the assessment of the capability of platelets to form a thrombus, which is important in hemostasis [52]. There was a nonsignificant difference between the nonmedicated aerogel (A_3_H_1_N) and the control (*p* > 0.05), whereas Cutanplast^®^ had a significantly lower recalcification time compared to the nonmedicated aerogel (*p* = 0.045). TXA significantly decreased the RT for both the TXA solution and A_3_H_1_ aerogel compared to the control (*p* ≤ 0.0001). Although the A_3_H_1_ aerogel had a shorter RT compared to the TXA solution, the difference was not significant (*p* = 0.44) (Figure 5A). The aerogel helped in preventing fibrin dissolution by entrapping it in the porous matrix (Figure 5B). The results also confirmed that TXA played the main role in fibrin formation and stabilization, owing to its antifibrinolytic activity [39]. The significant difference between the nonmedicated aerogel and Cutanplast^®^ may be attributed to Cutanplast^®^ being made of gelatin, which activates factor XII. This factor initiates the intrinsic pathway of the coagulation cascade [53].

#### 3.9.3. Platelet Adhesion

**Figure 6:**
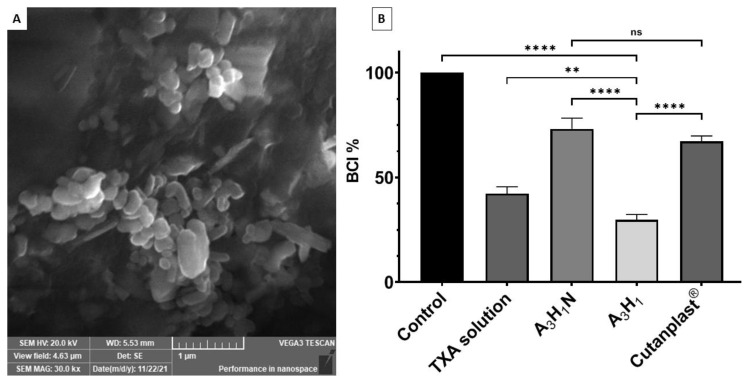
(**A**) Platelet adhesion to A_3_H_1_ aerogel matrix surface showing irregular shape of the platelets. (**B**) Blood clotting index of control sample, tranexamic acid solution, optimum aerogel with tranexamic acid (A_3_H_1_) and without tranexamic acid (A_3_H_1_N), and Cutanplast^®^; ns = *p* > 0.05, ** = *p* ≤ 0.01, and **** = *p* ≤ 0.0001.

After treatment of the A_3_H_1_ aerogel with PPP, the aerogel surfaces were covered with activated platelets (Figure 6A). The platelets had a polygonal irregular morphology on the matrix surface compared to its normal shape [54]. Uniform distribution of platelets along the matrix surface indicated complete adhesion of the platelets and the ability to initiate blood coagulation [55].

#### 3.9.4. In Vitro Dynamic Blood Clotting Index

A_3_H_1_N and A_3_H_1_ significantly reduced the BCI% relative to the control (*p* < 0.0001), with A_3_H_1_ significantly lower than A_3_H_1_N, as shown in Figure 6B. This test demonstrated the ability of the aerogel to stabilize the formed blood clot. Although blood coagulation took place in all samples, the strength of the produced clot is important. The A_3_H_1_N aerogel reduced the BCI by 27% compared to the control and it was not significantly different from Cutanplast^®^ (*p* = 0.1729). This might be due to entrapping the minimal number of RBCs and the formation of a weak fibrin network. This weak clot collapses by dilution with water, leading to the leakage and hemolysis of RBCs. For the A_3_H_1_ aerogel, only a few RBCs were hemolyzed, as shown by the low BCI% due to the antifibrinolytic effect of TXA that favors fibrin thread formation and strengthens the formed clot [34,56]. There was a significant decrease in BCI% for A_3_H_1_ compared to the TXA solution (*p* = 0.0033). This might be due to the added effect of both TXA and the hemostatic aerogel.

### 3.10. Swelling of the Optimum Aerogel in Blood

**Figure 7:**
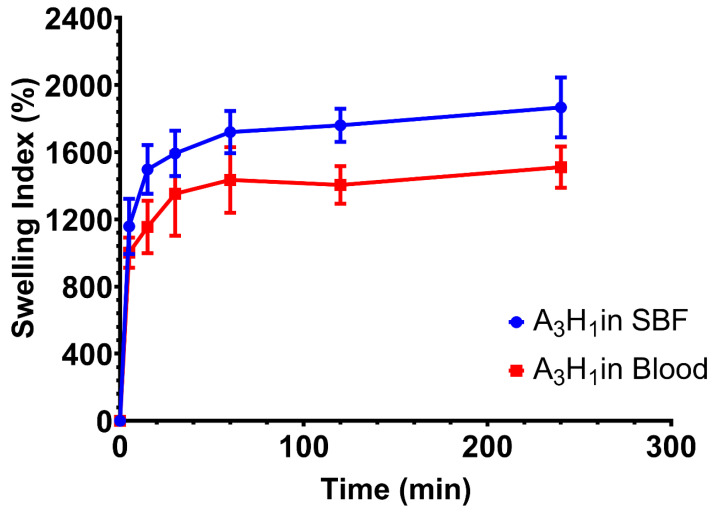
Swelling profile of A_3_H_1_ aerogel in simulated body fluid and blood.

The swelling behavior of the optimum aerogel (A_3_H_1_) in blood was tested and compared to that in the SBF. Results showed a similar trend of swelling over 4 h (Figure 7).

Swelling increased rapidly through the first 60 min, and then, it reached equilibrium. Statistical analysis of the swelling index of the aerogel in the two tested media at consecutive time points till 1 h showed nonsignificant differences (*p* > 0.05). The small difference between both profiles might be due to the viscous nature of blood and the fact that it is a cell-rich fluid. Both properties reduce the capacity of the aerogel to absorb blood into the matrix compared to a simple electrolyte solution such as SBF. However, swelling index values at the last two time points showed a significant difference (*p* < 0.05), reaching approximately 1511% in blood and 1867% in SBF after 4 h. This might be due to the formation of blood clots inside the matrix of the aerogel. These clots increased the rigidity of the matrix and blocked further absorption of fluids.

### 3.11. Indirect Cell Migration Assay

The wound healing assay is a typical in vitro technique for examining collective cell migration. In this assay, cells are removed via different mechanical or thermal damages to create a cell-free gap in a confluent monolayer. This induces cells to migrate into the gap [57].

**Figure 8:**
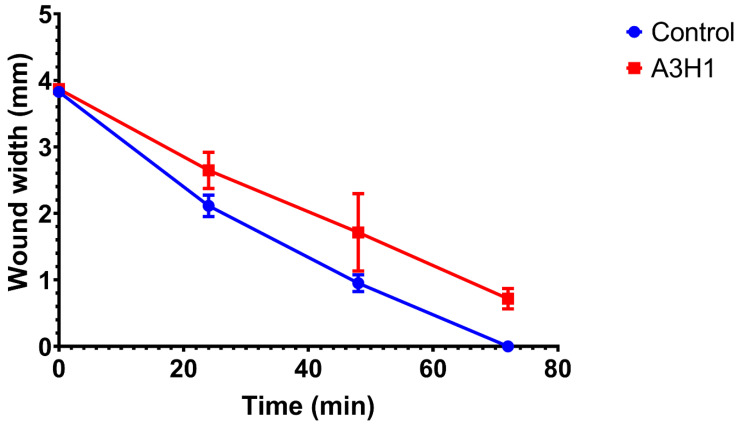
Cell migration test for control group and composite alginate/nano-hydroxyapatite aerogel loaded with tranexamic acid over 72 h.

**Figure 9:**
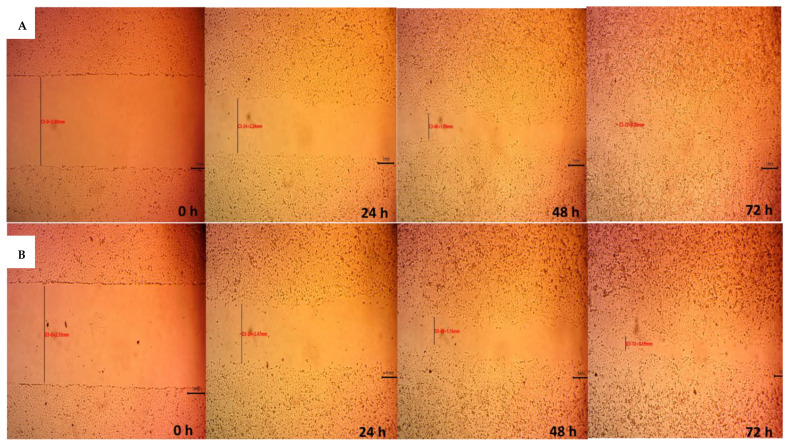
Microphotographs for cell migration assay of (**A**) control group, (**B**) alginate/nano-hydroxyapatite composite aerogel loaded with tranexamic acid over 72 h.

Figure 8 shows that the wound width decreases as a function of time for both the positive control group (cells in DMEM) and A_3_H_1_ aerogel extract. The migration rates were 0.052 ± 0.002 mm/h for the control group and 0.043 ± 0.003 mm/h for the aerogel. The wound gap closed completely after 68.7 ± 2.61 h for the control group and 87.3 ± 5.86 h for the aerogel. Figure 9 shows microphotographs of wound gap changes over 72 h. The wound width decreases gradually with time due to the fibroblasts’ collective migration to fill the gap. Statistical analysis showed that there was a significant difference between the migration rates of both test groups (*p* < 0.0001). This was in accordance with Wang et al. [58], who reported that the chronic exposure of fibroblasts to concentrations of TXA (12.5–25 mg/mL) may delay cell migration. In our work, a dose of 20 mg of TXA was used, and it delayed cell migration relative to the control group. This may be attributed to the cell detachment effect of TXA rather than cytotoxicity [59]. TXA binds to plasminogen to block its conversion to plasmin. Plasminogen was suggested to interact with integrin receptors that play a role in cell adhesion [60]. Consequently, altered cell adhesion and migration may occur. Although the composite aerogel loaded with TXA showed a good impact on hemostasis, it caused a slower wound healing process.

## 4. Conclusions

Tranexamic-acid-loaded alginate/nano-hydroxyapatite aerogels were successfully formulated to stop the bleeding following tooth extraction. The aerogels were highly porous in structure with a high swelling capability. These properties varied depending on the amounts of SA and Nano-HA in the aerogel. Aerogels devoid of Ca^+2^ ions in their backbone structure granted the fast release of TXA from their matrix, with a biphasic release profile extending to about 4 h. This provides an initial dose to rapidly stop the bleeding and a sustained dose to stabilize the formed clot. Blood coagulation studies confirmed the ability of the optimum aerogel to control bleeding through the formation of hydrogel-blood aggregates stabilizing the fibrin chains of the clot. Though further studies are needed in future work to evaluate the in vivo blood clotting action of alginate/nano-hydroxyapatite composite aerogels, we can conclude that this promising formulation could be considered as a successful local delivery system for the management of uncontrolled bleeding in patients on anticoagulant therapy and help in preventing alveolar osteitis.

## Figures and Tables

**Table 1:** Variables and responses with their levels and constraints for the 2^3^ factorial design.

Independent Variables	Levels	
X_1_: Concentration of sodium alginate	2% (*w*/*v*)	3% (*w*/*v*)
X_2_: Concentration of nano-hydroxyapatite	1% (*w*/*v*)	5% (*w*/*v*)
X_3_: Addition of CaCl_2_ solution	0	0.5 M
**Responses**	**Constraints**	
Y_1_: Porosity		High
Y_2_: Fluid uptake		High
Y_3_: Biphasic release profile		Exist

**Table 2:** Compositions of the prepared alginate/nano-hydroxyapatite composite aerogels loaded with tranexamic acid.

Aerogel Code	Factors Levels in Actual Values		
	X_1_: Concentration of Sodium Alginate (%)	X_2_: Concentration of Nano-Hydroxyapatite (%)	X_3_: Addition of CaCl_2_ Solution (M)
A_2_H_1_	2	1	-
A_2_H_5_	2	5	-
A_3_H_1_	3	1	-
A_3_H_5_	3	5	-
A_2_H_1_Ca	2	1	0.5
A_2_H_5_Ca	2	5	0.5
A_3_H_1_Ca	3	1	0.5
A_3_H_5_Ca	3	5	0.5

## Data Availability

The data presented in this study are available in the article and Supplementary Materials.

## Supplementary Materials

The following supporting information can be downloaded at: https://www.mdpi.com/article/10.3390/pharmaceutics14102255/s1, Figure S1: Alginate/Nano-hydroxyapatite composite aerogels loaded with tranexamic acid after lyophilization where (A) A_2_H_1_ [2% alginate and 1% Nano-hydroxyapatite],( B) A_3_H_1_ [3% alginate and 1% Nano-hydroxyapatite] (C) A_2_H_1_Ca [2% alginate crosslinked with 0.5 M CaCl_2_ and 1% Nano-hydroxyapatite], (D) A_3_H_1_Ca [3% alginate crosslinked with 0.5 M CaCl_2_ and 1% Nano-hydroxyapatite], (E) A_3_H_5_ [3% alginate and 5% Nano-hydroxyapatite], (F) A_2_H_5_ [2% alginate and 5% Nano-hydroxyapatite], (G) A_3_H_5_Ca [3% alginate crosslinked with 0.5 M CaCl_2_ and 5% Nano-hydroxyapatite], (H) A_2_H_5_Ca [2% alginate crosslinked with 0.5 M CaCl_2_ and 5% Nano-hydroxyapatite]. Figure S2: Effects of (A) alginate concentration, (B) nano-hydroxyapatite concentration, and (C) the addition of CaCl_2_ on the porosity of the prepared alginate/nano-hydroxyapatite composite aerogels loaded with tranexamic acid. Figure S3: Effects of (A) alginate concentration, (B) nano-hydroxyapatite concentration, and (C) the addition of CaCl_2_ on the swelling index of the prepared alginate/nano-hydroxyapatite composite aerogels loaded with tranexamic acid. Figure S4: Effects of (A) alginate concentration, (B) nano-hydroxyapatite concentration, and (C) the addition of CaCl_2_ on the release pattern of the prepared alginate/nano-hydroxyapatite composite aerogels loaded with tranexamic acid. Figure S5: FTIR spectra of (A) sodium alginate, (B) nano-hydroxyapatite, (C) tranexamic acid, (D) A_3_H_1_ aerogel. Table S1: Kinetics parameters of the prepared alginate/nano-hydroxyapatite composite aerogels loaded with tranexamic acid.
