# Supplementary material for: Hemostatic Alginate/Nano-Hydroxyapatite Composite Aerogel Loaded with Tranexamic Acid for the Potential Protection against Alveolar Osteitis
Source: Pharmaceutics. 2022 Oct 21;14(10):2255. doi: 10.3390/pharmaceutics14102255 (PMC9608763; doi:10.3390/pharmaceutics14102255)
Supplement: Supplementary file 1 [file pharmaceutics-14-02255-s001.zip › pharmaceutics-1895330-supplementary.pdf]

Hemostatic Alginate/Nano-Hydroxyapatite Composite Aerogel Loaded with Tranexamic Acid for the Potential Protection against Alveolar Osteitis

Mai El Halawany[1*], Randa Latif[1], Mohamed H.H. AbouGhaly[1,2]

[1]Department of Pharmaceutics and Industrial Pharmacy, Faculty of Pharmacy, Cairo University
[2]Department of Pharmaceutics and Industrial Pharmacy, School of Pharmacy, Newgiza University
*Corresponding author at: Department of Pharmaceutics and Industrial Pharmacy, Faculty of Pharmacy, Cairo University, Kasr El-Ainy Street, Cairo 11562, Egypt.
E-mail : mai.elhalawany@pharma.cu.edu.eg
Tel: +2 01008463596
Fax: +2 02 23628246

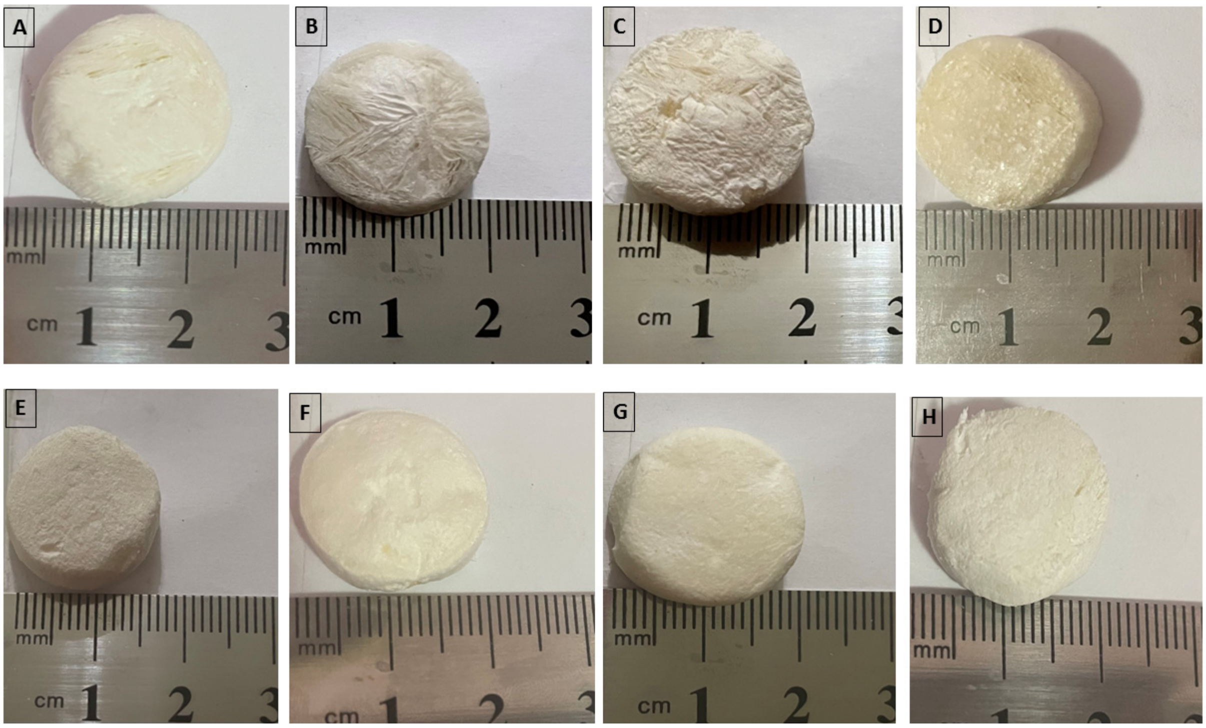

Figure S1: Alginate/Nano-hydroxyapatite composite aerogels loaded with tranexamic acid after lyophilization where (A) A_2H_1 [2% alginate and 1%Nano-hydroxyapatite], (B) A_3H_1 [3% alginate and 1%Nano-hydroxyapatite] (C) A_2H_1Ca [2% alginate crosslinked with 0.5M $CaCl_2$ and 1%Nano-hydroxyapatite], (D) A_3H_1Ca [3% alginate crosslinked with 0.5M $CaCl_2$ and 1%Nano-hydroxyapatite], (E) A_3H_5 [3% alginate and 5%Nano-hydroxyapatite], (F) A_2H_5 [2% alginate and 5%Nano-hydroxyapatite], (G) A_3H_5Ca [3% alginate crosslinked with 0.5M $CaCl_2$ and 5%Nano-hydroxyapatite], (H) A_2H_5Ca [2% alginate crosslinked with 0.5M $CaCl_2$ and 5%Nano-hydroxyapatite].

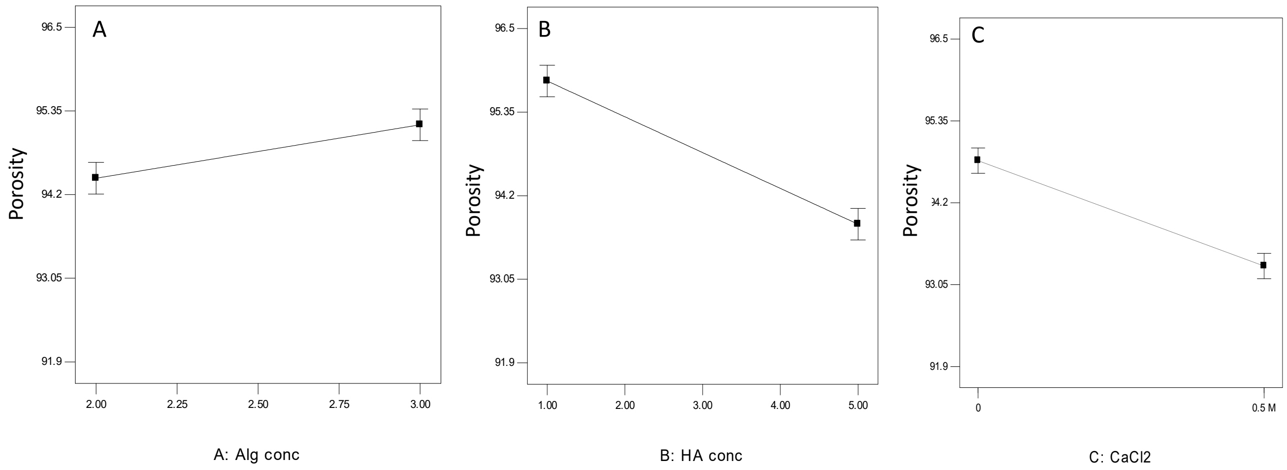

Figure S2: Effects of (A) alginate concentration, (B) nano-hydroxyapatite concentration, and (C) the addition of $CaCl_2$ on the porosity of the prepared alginate/nano-hydroxyapatite composite aerogels loaded with tranexamic acid.

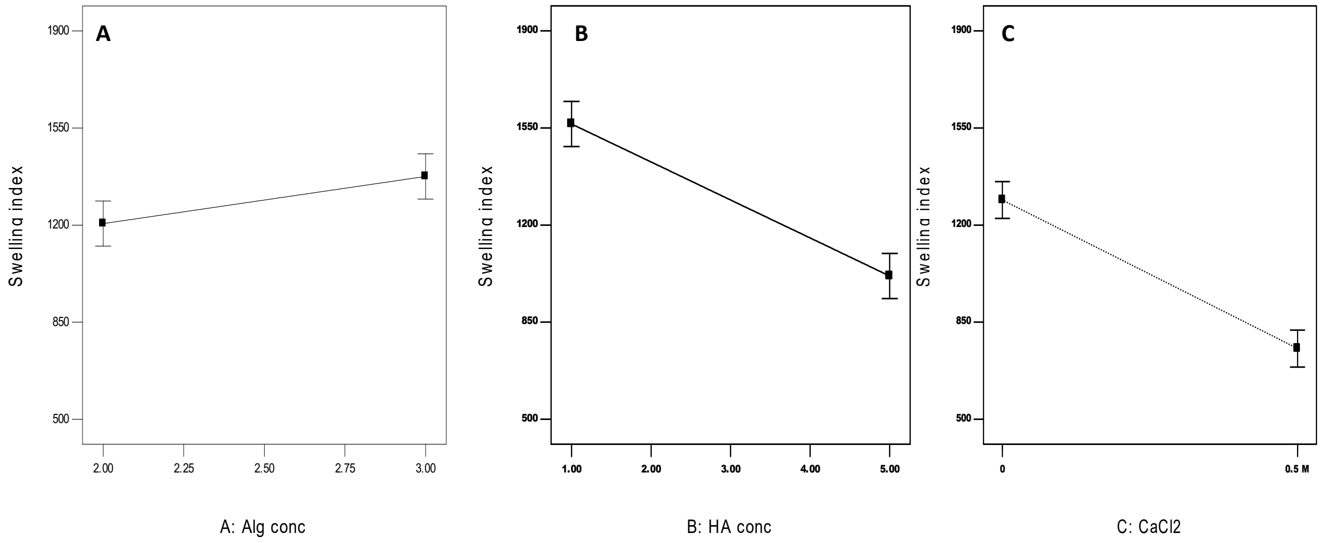

Figure S3: Effects of (A) alginate concentration, (B) nano-hydroxyapatite concentration, and (C) the addition of $CaCl_2$ on the swelling index of the prepared alginate/nano-hydroxyapatite composite aerogels loaded with tranexamic acid.

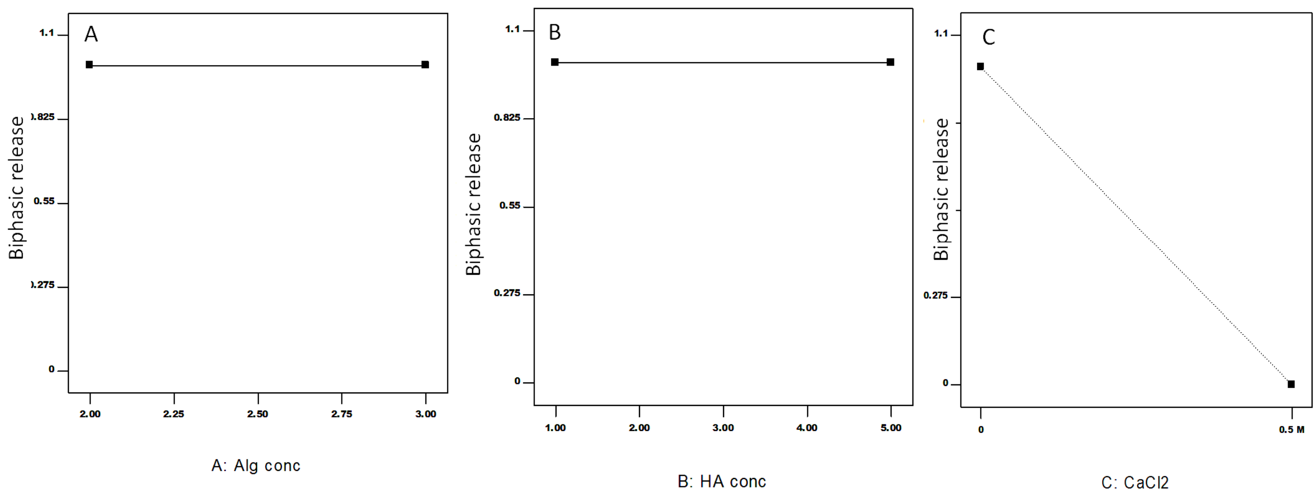

Figure S4: Effects of (A) alginate concentration, (B) nano-hydroxyapatite concentration, and (C) the addition of $CaCl_2$ on the release pattern of the prepared alginate/nano-hydroxyapatite composite aerogels loaded with tranexamic acid.

Table S1: Kinetics parameters of the prepared alginate/nano-hydroxyapatite composite aerogels loaded with tranexamic acid.

Code	Prepared aerogels with biphasic release profile											
	First phase (0 to 1 hr)						Second phase (1 to 4 hrs)					
	Zero order		First order		Higuchi diffusion		Zero order		First order		Higuchi diffusion	
	r^2	K (mg/min)	r^2	K (min^{-1})	r^2	K ($mg/min^{½}$)	r^2	K (min^{-1})	r^2	K ($mg/min^{½}$)	r^2	K ($mg/min^{½}$)
**A_2H_1**	0.945	0.782	0.988	0.017	0.991	8.16	0.989	0.126	0.933	0.011	0.963	1.658
**A_2H_5**	0.821	0.781	0.895	0.016	0.927	8.47	0.99	0.145	0.921	0.0108	0.926	1.85
**A_3H_1**	0.891	0.581	0.94	0.012	0.96	6.18	0.99	0.17	0.89	0.014	0.941	2.27
**A_3H_5**	0.919	0.752	0.976	0.015	0.98	7.94	0.937	0.155	0.812	0.0103	0.751	1.85

Code	Prepared aerogels with monophasic release profile					
	Zero order		First order		Higuchi diffusion	
	r^2	K (mg/min)	r^2	K (min^{-1})	r^2	K ($mg/min^{½}$)
**A_2H_1Ca**	0.932	1.169	0.953	0.087	0.963	12.129
**A_2H_5Ca**	0.959	0.556	0.911	0.05	0.997	7.706
**A_3H_1Ca**	0.891	0.529	0.967	0.033	0.979	7.532
**A_3H_5Ca**	0.931	0.519	0.975	0.028	0.989	7.261

Underlined numbers indicate highest Coefficient of determination (r^2), K: release rate constant

Fourier transform infrared spectroscopy

The spectrum of SA (Figure S5A) shows –OH stretching vibration at 3422 cm^{-1}, –CH stretching vibration at 2912 cm^{-1}, and asymmetric and symmetrical stretching vibration peaks of the carboxyl group at 1612 cm^{-1} and 1415 cm^{-1}, respectively. The C–O–C stretching vibrations appeared at 1029 cm^{-1}.

The spectrum of Nano-HA (Figure S5B) showed the bending peak of the phosphate group at 570 cm $^{-1}$. The O—P—O bending of the phosphate group appeared at 601 cm^{-1} and the asymmetric stretching band of the phosphate bonds (P—O) was observed at 1053 cm^{-1} [1].

The spectrum of TXA (Figure S5C) showed the CH_2 stretching wide band of aliphatic groups at 2990 and 2484 cm^{-1}. The C=O stretching of -COOH peak is seen at1664 cm^{-1}. The peaks at 1516 cm^{-1} can be assigned to the bending vibrations of the amino groups. The C-N in aliphatic amine peak is seen at 1006 cm^{-1} and the C-C bond of the carbon ring has a peak at 918 cm^{-1}. There are sharp absorption peaks at 767~524 cm^{-1}which are mainly caused by trans isomerism vibration [2,3].

The spectrum of A_3H_1 (Figure S5D) showed almost all absorption bands of the 3 components. Where, the wide peak at 3414 cm^{-1} was assigned to overlapping of surface hydroxyl groups and amino group that belongs to SA and TXA, respectively. Moreover, the observed bands about 1612 and 1387 cm^{-1} are attributed to symmetric and asymmetric stretching peaks of alginate carboxylate anions but they were slightly shifted and weakened because of partial crosslinking of Ca^{+2} ions to carboxylate groups [4]. The CH_2 stretching of aliphatic groups of TXA have been shifted slightly to 2935 cm^{-1}. The characteristic sharp peak at 1037 cm^{-1} was assigned to the combination of phosphate group stretching of Nano-HA and C–O–C stretching of SA and the bending peak of phosphate group in Nano-HA structure appeared at 570 cm^{-1}.

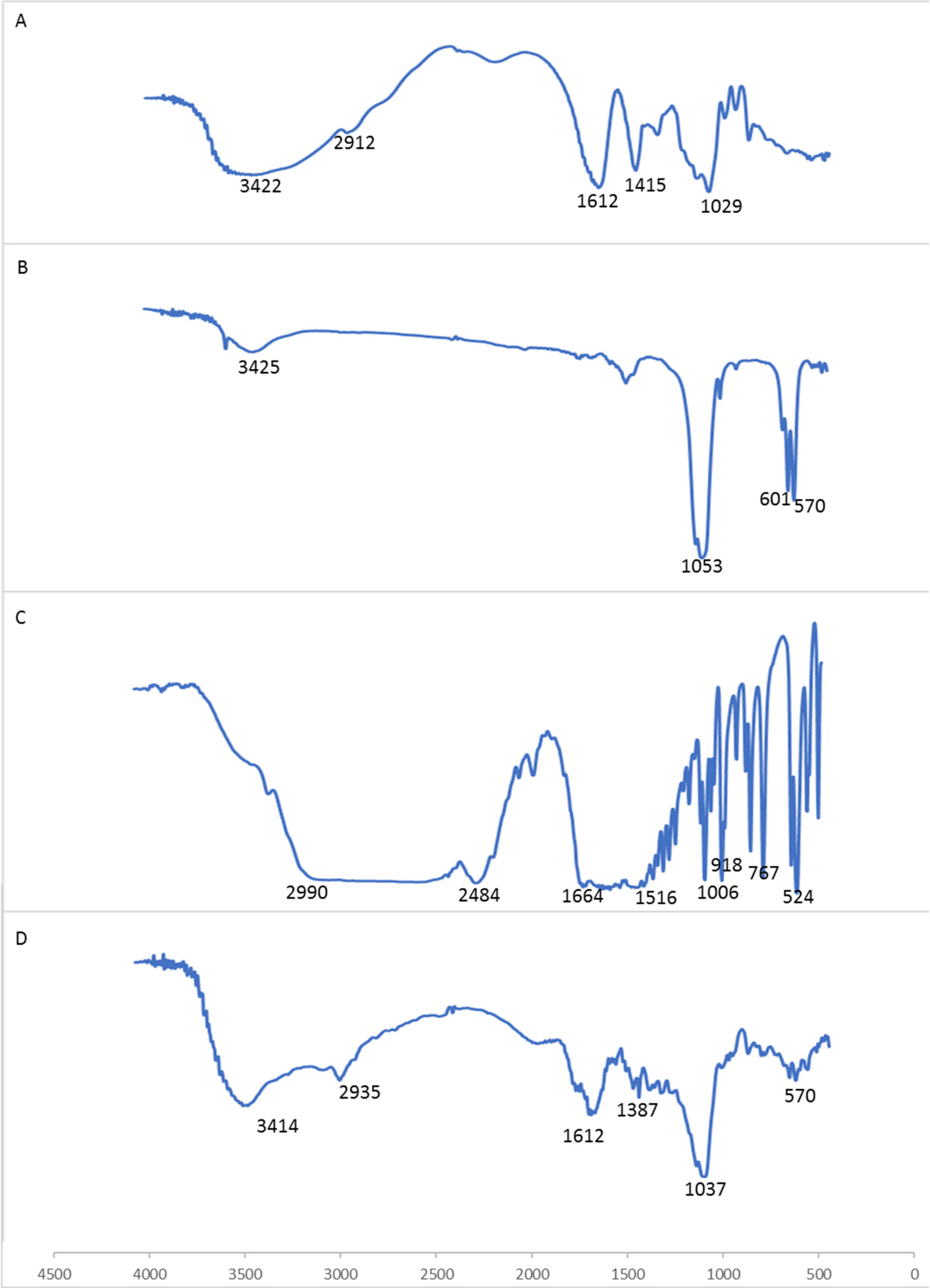

Figure S5: FTIR spectra of (A) sodium alginate, (B) nano-hydroxyapatite, (C) tranexamic acid, (D) A_3H_1 aerogel.

References

[1] Rajkumar, M.; Meenakshisundaram, N.; Rajendran, V. Development of nanocomposites based on hydroxyapatite/sodium alginate: Synthesis and characterisation. *Mater Character* **2011**, 62, 469-479, doi:10.1016/j.matchar.2011.02.008.

[2] Zhong, Q.-K.; Wu, Z.-Y.; Qin, Y.-Q.; Hu, Z.; Li, S.-D.; Yang, Z.-M.; Li, P.-W. Preparation and Properties of Carboxymethyl Chitosan/Alginate/Tranexamic Acid Composite Films. *Membranes* **2019,** 9,11, doi:10.3390/membranes9010011.

[3] Jin, H.-H.; Kim, D.-H.; Kim, T.-W.; Shin, K.-K.; Jung, J.S.; Park, H.-C.; Yoon, S.-Y. In vivo evaluation of porous hydroxyapatite/chitosan–alginate composite scaffolds for bone tissue engineering. *Int J Biol Macromol* **2012**, *51*, 1079-1085, doi:10.1016/j.ijbiomac.2012.08.027.

[4] Gholizadeh, B.S.; Buazar, F.; Hosseini, S.M.; Mousavi, S.M. Enhanced antibacterial activity, mechanical and physical properties of alginate/hydroxyapatite bionanocomposite film. *Int J Biol Macromol* **2018**, *116*, 786-792, doi:10.1016/j.ijbiomac.2018.05.104.
